# A general skull stripping of multiparametric brain MRIs using 3D convolutional neural network

**DOI:** 10.1038/s41598-022-14983-4

**Published:** 2022-06-27

**Authors:** Linmin Pei, Murat Ak, Nourel Hoda M. Tahon, Serafettin Zenkin, Safa Alkarawi, Abdallah Kamal, Mahir Yilmaz, Lingling Chen, Mehmet Er, Nursima Ak, Rivka Colen

**Affiliations:** 1 Imaging and Visualization Group, ABCS, Frederick National Laboratory for Cancer Research, Frederick, MD 21702 USA; 2 Department of Radiology, University of Pittsburgh, Pittsburgh, PA 15260 USA; 3 Hillman Cancer Center, University of Pittsburgh Medical Center, Pittsburgh, PA 15232 USA

**Keywords:** Computational models, Cancer imaging

## Abstract

Accurate skull stripping facilitates following neuro-image analysis. For computer-aided methods, the presence of brain skull in structural magnetic resonance imaging (MRI) impacts brain tissue identification, which could result in serious misjudgments, specifically for patients with brain tumors. Though there are several existing works on skull stripping in literature, most of them either focus on healthy brain MRIs or only apply for a single image modality. These methods may be not optimal for multiparametric MRI scans. In the paper, we propose an ensemble neural network (EnNet), a 3D convolutional neural network (3DCNN) based method, for brain extraction on multiparametric MRI scans (mpMRIs). We comprehensively investigate the skull stripping performance by using the proposed method on a total of 15 image modality combinations. The comparison shows that utilizing all modalities provides the best performance on skull stripping. We have collected a retrospective dataset of 815 cases with/without glioblastoma multiforme (GBM) at the University of Pittsburgh Medical Center (UPMC) and The Cancer Imaging Archive (TCIA). The ground truths of the skull stripping are verified by at least one qualified radiologist. The quantitative evaluation gives an average dice score coefficient and Hausdorff distance at the 95th percentile, respectively. We also compare the performance to the state-of-the-art methods/tools. The proposed method offers the best performance.

The contributions of the work have five folds: first, the proposed method is a fully automatic end-to-end for skull stripping using a 3D deep learning method. Second, it is applicable for mpMRIs and is also easy to customize for any MRI modality combination. Third, the proposed method not only works for healthy brain mpMRIs but also pre-/post-operative brain mpMRIs with GBM. Fourth, the proposed method handles multicenter data. Finally, to the best of our knowledge, we are the first group to quantitatively compare the skull stripping performance using different modalities. All code and pre-trained model are available at: https://github.com/plmoer/skull_stripping_code_SR.

## Introduction

In the U.S., there were 23 per 100,000 population diagnosed with brain tumors during 2011–2015^1^. Gliomas, originate from glial cells, are the most common primary brain malignancies, with varying degrees of aggressiveness^2^. To make a proper treatment planning, accurate brain tumor detection and segmentation are strongly demanding. Due to time-consuming, inter-rater prone error, and low efficacy, manual brain tumor segmentation by radiologists is very challenging, and is not feasible for large-scale data^3^. Therefore, an automatically computer-aided brain tumor segmentation/detection is highly desired^3–9^. However, a high-resolution brain magnetic resonance image (MRI) contains non-brain tissues, such as eyeball, skin, neck, skin, and muscle^10^. The presence of the non-brain tissues is one of the major challenges for automatic brain image analysis. The non-brain tissues removal is a typical preprocessing step for most brain MRI studies, e.g., brain volumetric measurement^11^, brain tissue segmentation^12^, assessing schizophrenia^13^, and Alzheimer’s disease^14^. Consequently, before applying automatic computational technique for brain MRI studies, skull stripping is a prerequisite for brain imaging analysis^15^.

As a preprocessing step, skull stripping (i.e., brain extraction) removes the skull and other non-brain tissues out from the MRI scans. It reduces human rater variance and eliminates time-consuming manual processing steps that potentially impede not only the analysis but also the reproducibility of large-scale studies^16^. The quality of skull stripping can be affected by several reasons, including imaging artifacts, MRI scanners, and acquisition protocol, etc. Furthermore, variability of anatomy, age, and the extent of brain atrophy, has impact on skull stripping as well^17^. When considering MRI scans with pathological conditions, such as brain tumors, the problem becomes more complicated. Intensity of brain tissues in MRI may be impacted due to presence of brain tumor. The situation could become worse when dealing with post-treatment of the MRI with brain tumors, specifically with resection surgery. The cavities resulting from resection not only change the reflection of intensity but also alter the brain anatomy. All these factors above undermine the performance of skull stripping.

We argue that a good skull stripping leads to a good following-up brain analysis. Therefore, in the paper, we propose a 3D deep neural network-based method for skull stripping. The proposed method utilizes multiparametric MRIs for skull stripping. Different MR acquisition protocols provides complementary information about brain tissues, which facilitates a better separation between brain, cerebrospinal fluid (CSF), and other tissues, such as skull, or fat. With ensemble of the high dimensional features by using the proposed method, the integration of all multiparametric MRI sequences offers the highest accuracy of brain extraction.

The contributions of this work include: first, it is a fully automatic end-to-end technique for skull stripping using a 3D deep learning method; second, it is applicable for multiparametric MRI (mpMRIs) and is also easy to customize for a single MRI modality; third, it works not only for healthy brain MRI, but also for pre-/post-operative brain MRI with a brain tumor; fourth, the proposed method applies to multicenter data; finally, as the best of our knowledge, we are the first group to quantitatively compare the skull stripping performance using different modalities.

## Previous work

There are several skull stripping methods proposed in literature. These methods can be broadly classified into four categories: morphology-based, intensity-based, deformable surface-based, and atlas-based^10^. The morphology-based methods utilize a morphological erosion and dilation operations to remove skulls from the brain. Brummer et al*.* proposed an automatic skull stripping on MRI using a morphology-based method^18^. It combines histogram-based thresholding and morphological operations for skull stripping. Similar work presented in^19^, authors performed a 2D Marr-Hildreh operator to achieve edge detection, then employed several morphological operations for skull stripping. However, it is difficult to find the optimal morphology-based method. In addition, the proposed methods are sensitive to small data variations. Proper thresholding and edge detection are the challenges for these methods. For intensity-based methods, they separate the brain and non-brain according to the image intensity. A typical technique of the method is a watershed algorithm. The watershed algorithm extracts foreground and background, and then uses markers to make watershed run and detect the exact boundaries. Hahn et al. utilized the watershed algorithm to remove skull on T1-weighted MR images^20^. There are some similar works, such as^21,22^. These methods depend on the correctness of intensity distribution modeling and are sensitive to intensity bias. The deformable surface-based methods evolve and deform an active contour to fit the brain surface. A popular tool named the Brain extraction tool (BET) employs a deformable model for separating brain and non-brain from MRI^23^. BET2 is the extension of BET, which generates a better result based on a pair of T1- and T2-weighted MRI^24^. Other work, such as^25,26^ also use the deformable surface-based methods for the skull stripping. However, these methods rely on the location of the initial curve and the image gradient^10^. The atlas-based methods use the transferring knowledge of the anatomical structure of a template to separate skull and brain, such as work^27,28^. Roy et al. proposed a robust skull stripping which uses a sparse patch based Multi-cONtrast brain STRipping method (MONSTR)^29^. However, these methods rely on specific image modalities and lack flexibility of extending to other modalities, such as the work in^29^ limits to the T1w and T2 modalities.

These atlas-based methods highly rely on the quality of image registration, and they are suffering from for multiparametric MRIs. Moreover, they are not applicable for pathological MRIs which contain brain tumors/diseases.

In recent years, because of computer hardware development and big data availability, deep learning has been becoming prevalent in many domains, such as image analysis^30,31^, natural language processing (NLP)^32^, computer vision^33^, speech recognition^34^, etc. Deep learning-based methods are also applied to medical image analysis, including brain segmentation^35^, brain tumor classification^36^, brain tumor segmentation^7^, and lung cancer segmentation^37^, etc. Deep learning-based methods also apply for skull stripping, such as^16,38–40^. However, these methods may either only apply for normal healthy brain skull stripping, pre-operative brain with gliomas, or difficultly extend to other image modalities. We believe that there still are many spaces to improve the skull stripping performance by employing advanced deep-learning based methods. Therefore, to overcome the limitations mentioned above, we propose a 3D convolutional neural network (3DCNN)-based end-to-end method for a general skull stripping. It not only works for healthy brain MRIs, but also for pre-/post-operative brain MRIs with glioblastoma multiforme (GBM). Furthermore, it is applicable for multicenter data.

## The proposed method

All experiments in this study are performed in accordance with relevant guidelines and regulations as approved by the institutional IRB committee at the University of Pittsburgh. Approval was obtained from the ethical committee of University of Pittsburgh (Study19119234: PanCancer Imaging and Imaging Genomic Analysis).

Deep neural networks have been becoming successful in many domains and achieve state-of-the-art performance for many applications. Therefore, in the work, we build a deep neural network-based method for skull stripping because of its advantages. The motivation for creating a novel skull stripping has three facets. The first one is to process multiparametric brain MRI (mpMRI), which includes T1-weighted (T1), T1-weighted and contrast-enhanced (T1ce), T2-weighted (T2), and T2-fluid-attenuated inversion recovery (T2-FLAIR). The mpMRI offers a better result of skull stripping than that of a single image sequence. Moreover, it is easy to customize for any image sequence combination. Last, the proposed method is general for all conditional cases, including healthy brain MRI, and pre-/post- operative brain MRI.

**Figure 1:**
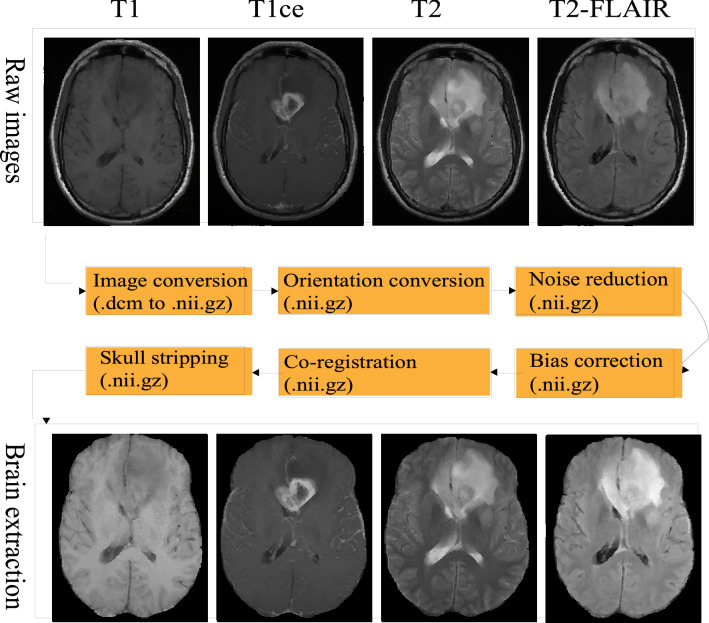
The whole workflow of brain extraction proposed in this work.

The whole workflow of brain extraction is shown in Fig. 1. Firstly, we convert the raw digital imaging and communication in medicine (.dicom) multiparametric images into a compressed neuroimaging informatics technology initiative (.nii.gz) format, then change the orientation same as to the SRI24 atlas^41^. There are then two optional pre-processing steps: noise reduction and bias field correction. Subsequentially, each imaging modality registers to the atlas (1 mm × 1 mm × 1 mm), so that all image modalities are aligned into the same space having resolution of 1 mm × 1 mm × 1 mm. Thereafter, all co-registered isotropic image modalities are stacked following the sequence of T2-FLAIR, T1, T1ce, and T2. Finally, the fused images (dimension: $4\times 155\times 240\times 240$) are fed into the proposed deep neural network model to obtain a binary mask for skull stripping. The co-registered brain extraction is accomplished by multiplying the binary mask to the co-registered images.

**Figure 2:**
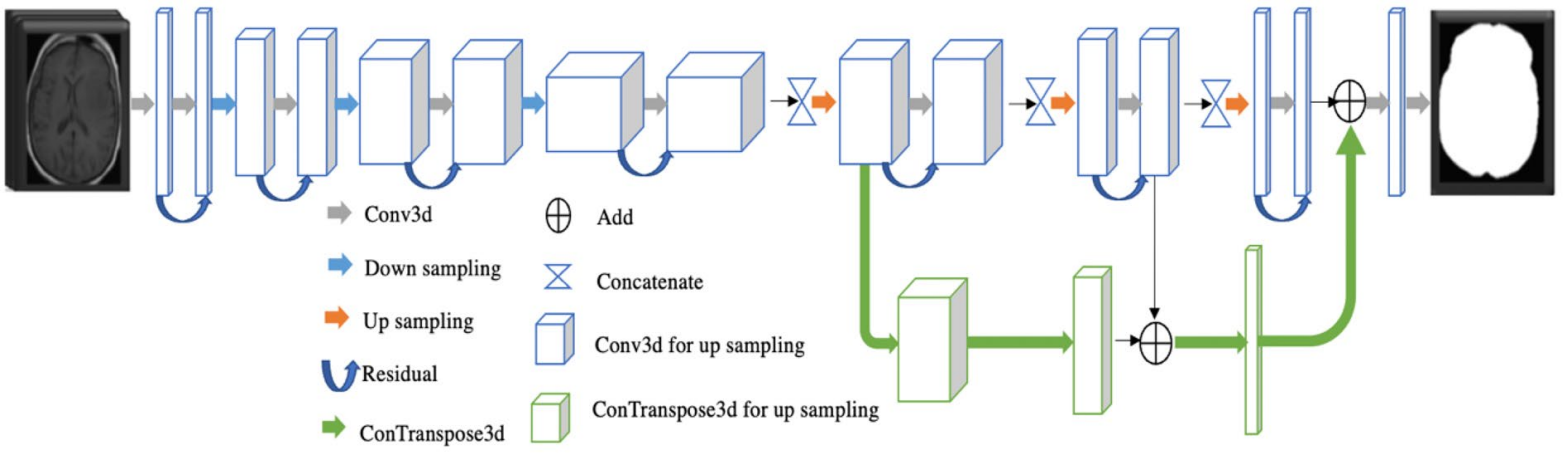
The proposed deep neural network architecture for skull stripping. The blue and green blocks represent feature maps from two different convolutional operations. The former features are obtained from a conv3d computation, while the latter from a conTranspose3d operation. The gray arrow represents a conv3d computation.

The proposed architecture of a deep neural network is illustrated in Fig. 2*.* The proposed architecture customizes the existing UNet^42^ with a branch of feature ensemble. There are two main parts of the network. The first encoder part is to extract high-dimensional features. The encoder part consists of several convolution blocks and max-pooling blocks. A convolution block is composed of convolution with residual connection, group normalization, and leaky rectified linear unit. Another part is a decoder, which is the opposite function to the encoder. The decoder expands the high-dimensional features to the target segmentation. It consists of convolution blocks and up-sampling blocks. In addition, we design an extra block (convolution block in green). We ensemble the feature maps by adding features from the regular decoder and that of the additional decoder. The feature maps aim to enforce the training convergence. We name the proposed architecture as an ensemble neural network (EnNet). For each residual block, it contains two convolutional layers, two group normalizations (GroupNorm), and two leaky Relu layers. For implementation details, please refer to source files in git repository at https://github.com/plmoer/skull_stripping_code_SR.

## Materials and experiment

### Dataset

In this work, we use a total of 815 cases (347 female cases and 468 male cases) from multi-center for the experiment. Age of all patients ranges from 28 to 93. Each case has mpMRIs which contain T1-weighted (T1), T1-weighted and contrast-enhanced (T1ce), T2-weighted (T2), and T2-fluid-attenuated inversion recovery (T2-FLAIR). Within the 815 cases, 776 cases are obtained from the University of Pittsburgh Medical Center (UPMC), and the rest of 39 cases are coming from The Cancer Imaging Atlas (TCIA), which collects data from multiple institutes. The data distribution is listed in Table 1. In UPMC, the mpMRIs are acquired from three different GE Healthcare System platforms: Discovery MR750 (3 T), Optima MR450W (1.5 T), and Signa HDxt (1.5 T). Unfortunately, we cannot find the device information for mpMRIs from TCIA dataset. The image size varies from $256\times 256\times 23$ to $512\times 512\times 89$, where 23 and 89 is the slice number of each case. For the atlas, the size of the SRI24 is $240\times 240\times 155$.

**Table 1:** Data distribution in the experiment.

Phase	# of case	Center	MRI status
Training	480	UPMC	Pre-operative
Validation	119	UPMC	Pre-operative
Testing	216	UPMC (177 cases)	Pre-operative (57 cases)
			Post-operative (57 cases)
			Healthy (63 cases)
		TCIA (39 cases)	Pre-operative (20 cases)
			Post-operative (19 cases)

### Experiment setup

Before skull stripping, there are several pre-processing steps, including image format conversion, orientation change, noise reduction, bias correction, and co-registration, as details discussed in Section III. In the experiment, all cases are randomly split into training, validation, and testing dataset with ratio of 0.6:0.15:0.25. Specifically, there are 480 cases, 119 cases, and 216 cases for training, validation, and testing dataset, respectively. In the testing dataset, there are 177 cases and 39 cases from UPMC and TCIA, respectively. More specially, the 177 cases consist of 57, 57, and 63 cases for normal brain, pre-operative, post-operative cases, respectively. The 39 TCIA cases are composed of 20 pre-operative and 19 post-operative MRIs. Note that the training and validation data are obtained from our in-house UPMC, but the testing cases are obtained from both UPMC and TCIA for evaluating the generality of the proposed method.

The proposed EnNet is implemented using Pytorch (version 1.10.0). We execute the algorithm on a Nvidia Titan X with 12 GB RAM with the operating system Linux. To prevent overfitting and improve the generalization capacity of the model, data augmentation is applied on the fly during training process. It includes random crop 3D, random rotation (0, 10°), random intensity change (− 0.1, 0.1), and random flip.

### Hyper-parameter setting

In each iteration, we randomly crop all co-registered MRIs with the size of 160 × 192 × 128 because of the limited capacity of the graphics processing unit (GPU). We believe that the cropped image covers the most region-of-interest (ROI). The epoch number is 300 for the training process. The batch size is set as 1 due to the large patch size and limited GPU memory. The loss function is computed as follows:

1 $$L=1-\frac{2\times \sum p\times \sum y}{\sum {p}^{2}+\sum {y}^{2}+\epsilon }$$

where $p$ and $y$ are the class prediction and ground truth (GT) at each voxel, respectively. The $\epsilon$ is a very small value.

Even the Adam^43^ optimizer has poor generalization ability, it converges faster than stochastic gradient descent with momentum (SGDM)^44^. Therefore, Adam is widely used in deep learning-based digital image application. In the experiment, we employ the Adam optimizer with an initial learning rate of $l{r}_{0}=0.001$ in training phase, and the learning rate (${r}_{i}$) is gradually decayed by the following:

2 $${r}_{i}= {r}_{0}{*(1-\frac{i}{N})}^{0.9},$$

where $i$ is an epoch counter, and $N$ is the total number of epochs in training.

### Evaluation measurements

To quantitatively evaluate the performance of the proposed method, we employ several evaluation metrics in the work, such as dice, precision, recall, false positive rate (FPR), false negative ration rate (FNR), and Hausdorff distance at the 95 percentiles (HD95). They are calculated as follows:

3 $$\mathrm{Dice}=\mathrm{F}= \frac{\mathrm{TP}}{2\mathrm{TP}+\mathrm{FN}+\mathrm{FP}}$$

4 $$\mathrm{Precision}= \frac{\mathrm{TP}}{\mathrm{TP}+\mathrm{FP}}$$

5 $$\mathrm{Recall}= \frac{\mathrm{TP}}{\mathrm{TP}+\mathrm{FN}}$$

6 $$\mathrm{FPR}= \frac{\mathrm{FP}}{\mathrm{FP}+\mathrm{TN}}$$

7 $$\mathrm{FNR}= \frac{\mathrm{FN}}{\mathrm{FN}+\mathrm{TP}}$$

8 $$\mathrm{HD}95=\mathrm{percentile}({\mathrm{max}}_{\mathrm{a\epsilon pred}}{\mathrm{min}}_{\mathrm{b\epsilon gt}}\{\mathrm{d}(\mathrm{pred},\mathrm{ gt})\} , {95}^{\mathrm{th}} )$$

where TP, FN, FP, TN are true positive, false negative, false positive, and true negative, respectively.

Dice is a statistic matrix that measures the similarity of the prediction and ground truth^45^. A value of 1 means that the two groups are identical, and a value of 0 shows no overlap at all between the two groups. The precision indicates how many of the positively classified are relevant. Recall, also known as sensitivity, represents how good a test is at detecting the positives. The Hausdorff distance (HD) measures the extent to which each point of a model set (prediction) lies near some points of an image set and vice versa^46^. A smaller value of HD suggests more similarity.

## Results

In the section, we first share the overall performance of skull stripping using the proposed method, then investigate the performance difference for several conditional MRIs (healthy brain MRIs, pre-operative brain MRIs, and post-operative brain MRIs), subsequentially estimate the model robustness across multicenter data, and finally compare with state-of-the-arts.

### Overall performance of skull stripping

As of the combination of all image sequences provides the best performance, we employ the best model for the testing data in the testing phase. With the total number of 216 testing cases, our algorithm offers an average dice of $0.9851\pm 0.017$. The complete evaluation metrics are shown in Table 2*.*

**Table 2:** Overall performance of skull stripping in the testing phase.

	Dice	Precision	Recall	FPR	FNR	HD95 (mm)
Ave	0.9850	0.9940	0.9768	0.0012	0.0232	2.6098
Std	0.0171	0.0093	0.0307	0.0019	0.0307	2.4814

### Generality of the model

**Figure 3:**
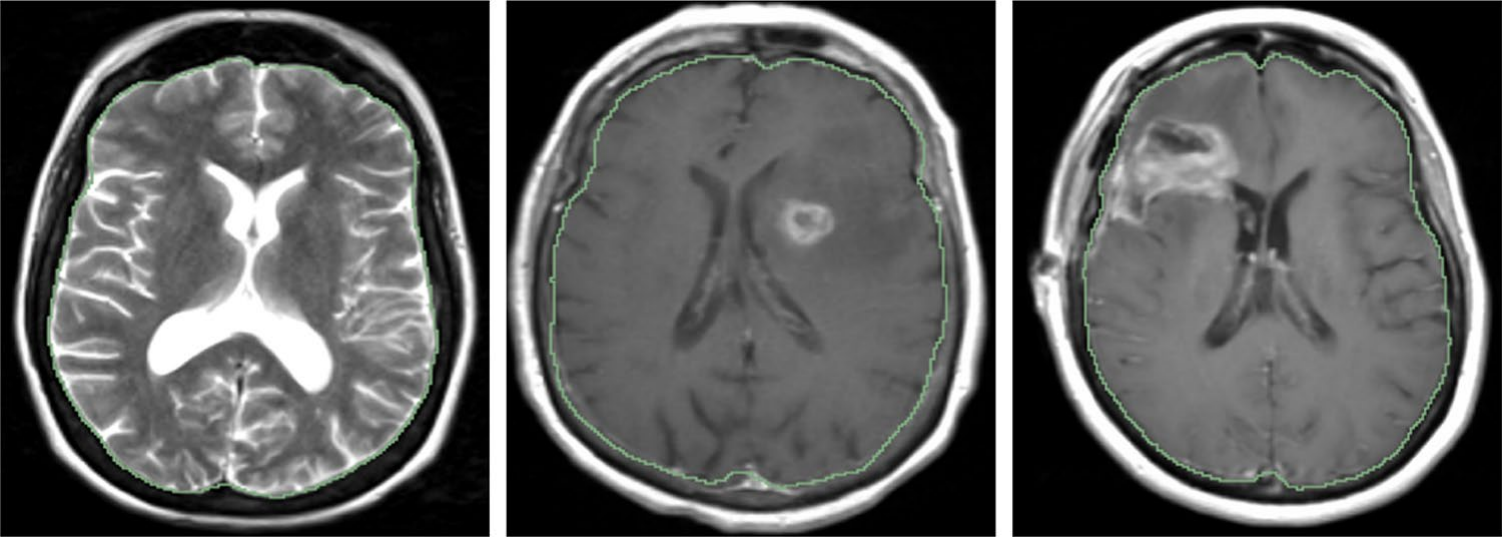
Showcases of skull stripping in different stage: healthy brain T2-weighted MRI (left), pre-operative T1-ce brain tumor MRI (middle), and post-operative T1-ce brain tumor MRI (right). The green contour is the boundary of skull stripping using the proposed method.

As discussed early, the proposed method works not only in healthy brain MRIs, but also in pre-/post- operative MRIs. To quantitatively evaluate the performance difference, we set up an experiment. The result is shown in Table 3. Interestingly, we noticed that the best results happened in pre-operative brain tumor MRIs, rather than in healthy brain MRIs. In addition, we also compute the t-test among different types of MRI, as shown in Table 3. The p-value of Healthy vs. Post-op, Healthy vs. Pre-op, and Pre-op vs. Post-op is 0.3869, 0.0204, and 0.2301, respectively. It indicates a significant performance difference between healthy and pre-operative MRIs, but no significant difference in rest cases. The reason may be that the training data is from the pre-operative mpMRIs with glioblastoma. Overall, the skull stripping performance is stable in all conditions, either the healthy brain MRIs, or brain tumor MRIs. There are 3 showcases shown in Fig. 3.

**Table 3:** Performance comparison of skull stripping for different stage MRIs of UPMC data.

Type of MRI	# of cases	Dice	Precision	Recall	FPR	FNR	HD95 (mm)	p-value (t-test)
Healthy MRIs (Healthy)	57	0.9851 ± 0.0139	**0.9963** ± 0.0070	0.9745 ± 0.0260	**0.0007** ± 0.0013	0.0255 ± 0.026	2.4399 ± 1.568	0.3869 (vs Post-op)
Pre-operative MRIs (Pre-op)	57	**0.9906** ± 0.0068	0.9910 ± 0.0111	**0.9904 ± **0.0097	0.0019 ± 0.0027	**0.0096** ± 0.0097	**2.1655** ± 1.5278	**0.0204 (vs Healthy)**
Post-operative MRIs (Post-op)	63	0.9894 ± 0.0213	0.9942 ± 0.0080	0.9852 ± 0.0358	0.0011 ± 0.0016	0.0148 ± 0.0358	2.1751 ± 3.6873	0.2301 (vs Pre-op)

The best result is highlighted in bold.

## Model robustness across multicenter

It is common that brain MRIs are acquired from multiple centers/institutes using different acquisition machines or following different protocols. The multicenter issue may undermine the performance of a model training with a single-center data. In this work, we also investigate the model robustness across multicenter. Additional to our in-house UPMC data (177 cases), we randomly take 39 cases (20 pre-operative cases and 19 post-operative cases) from TCIA that collects MRIs datasets from multiple institutes/hospitals. The experimental result is summarized in Table 4. We further calculate the t-test between the two data sources, the p-value is 0.0306, which shows a significant performance difference.

**Table 4:** Skull stripping performance across multicenter (UPMC and TCIA).

Center	# of cases	Dice	Precision	Recall	FPR	FNR	HD95 (mm)	p-value (t-test)
UPMC	177	**0.9884** ± 0.0155	0.9939 ± 0.0091	**0.9834** ± 0.0272	0.0012 ± 0.002	**0.0166** ± 0.072	**2.2573** ± 2.516	**0.0306**
TCIA	39	0.9699 ± 0.0016	**0.9946** ± 0.0105	0.9409 ± 0.0281	**0.0010** ± 0.0018	0.0531 ± 0.0281	4.2099 ± 1.52	–

The best result is highlighted in bold.

The comparison of the summary indicates that the performance at TCIA is around 2% lower than that of data obtaining from the same center for model training. However, the skull stripping performance across multicenter achieves good enough for following medical image analysis.

### Comparison of state-of-the-art

**Figure 4:**
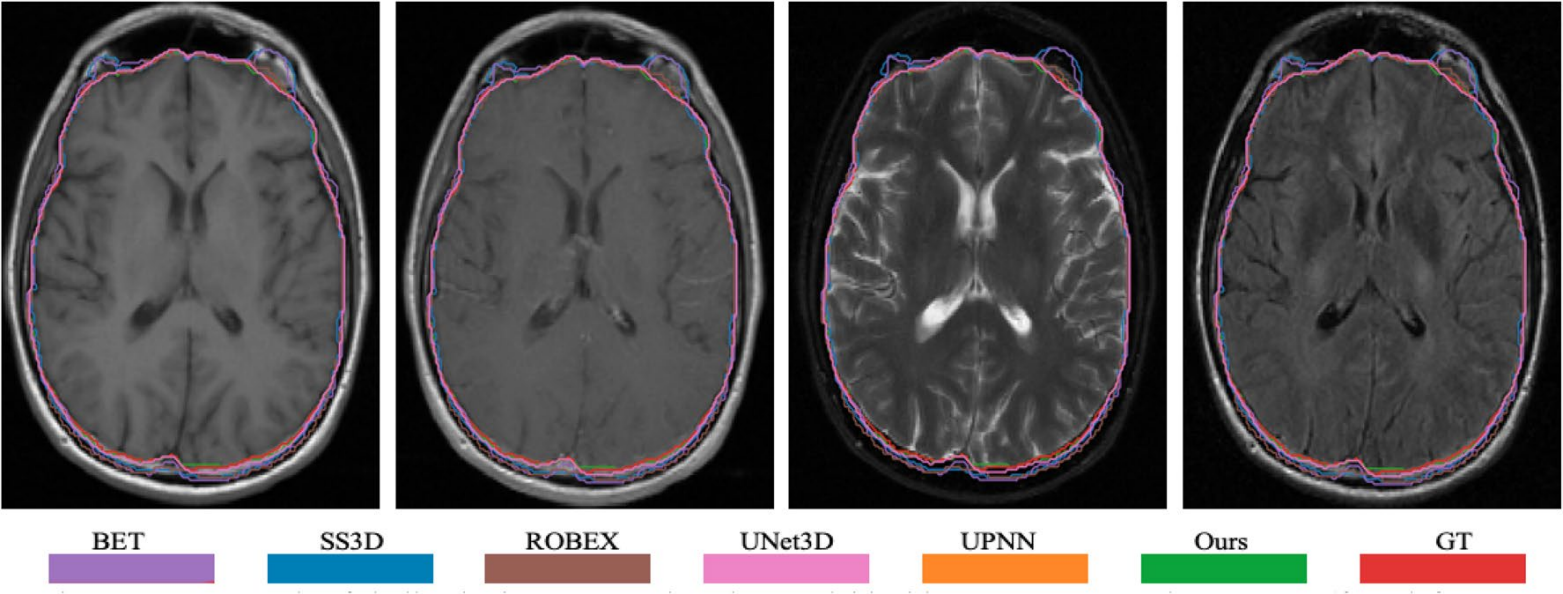
An example of skull stripping contours in color overlaid with T1, T1ce, T2, and T2-FLAIR (from left to right) using different methods/tools (color image for a better visualization). GT is the ground truth.

**Figure 5:**
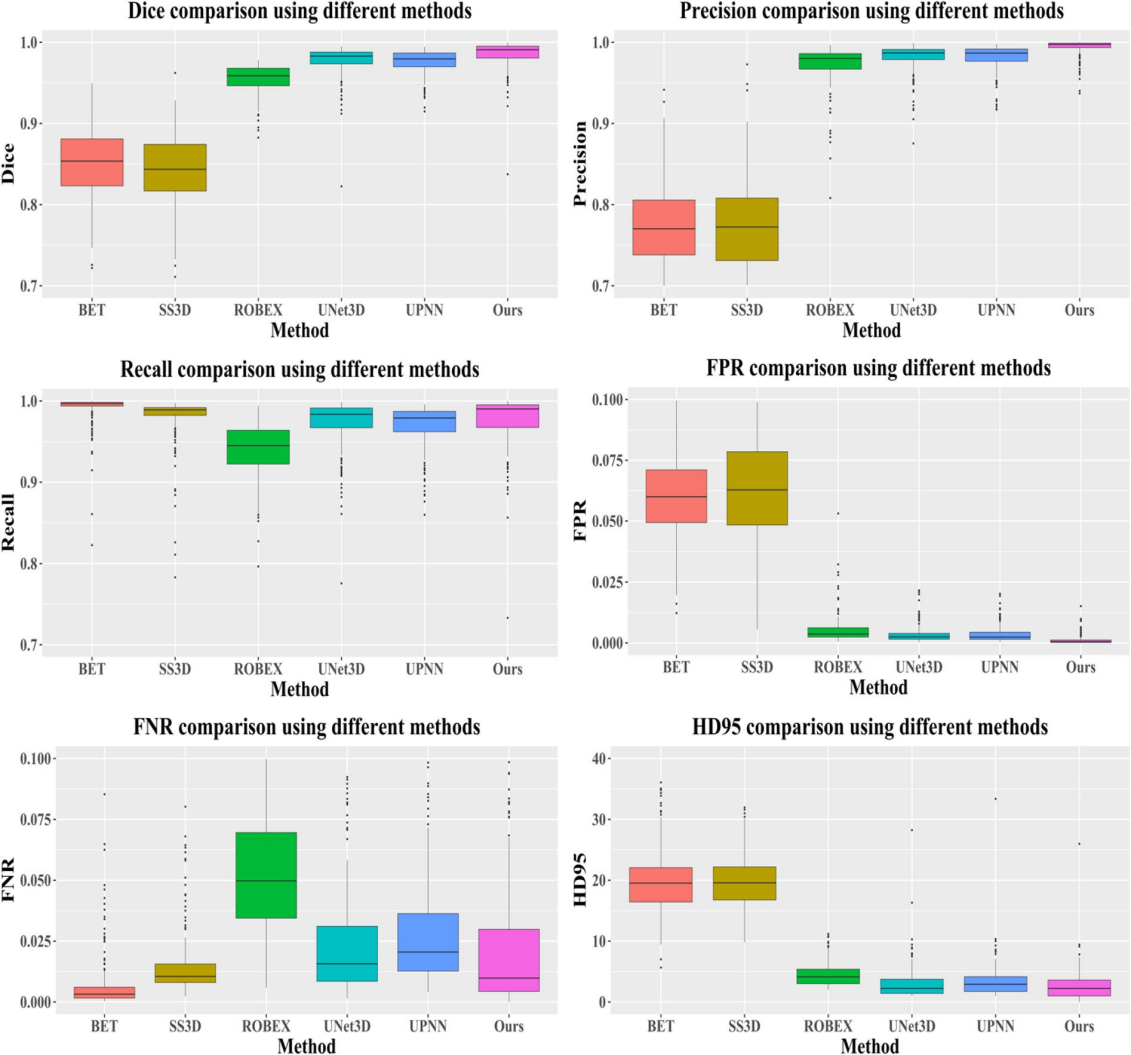
Box plot of performance comparison to state-of-the-arts on dice (top left), precision (top right), recall (middle left), FPR (middle right), FNR (bottom left), and HD95 (bottom right), respectively.

In the work, we also compare the performance of skull stripping using the proposed deep learning-based method to the popular methods/tools. The selectively popular tools include three traditional computer vision-based methods and two deep learning-based methods. In doing so, we either re-implement the algorithm or directly use the published tools. The popular methods/tools include Brain Extraction Tool (BET)^23^, 3d skull stripping (3dSS)^47^, Robust Learning-Based Brain Extraction (ROBEX)^48^, UNet 3D (UNet3D)^38^, and DeepMedic by UPNN^39^. The main difference between the two deep learning-based methods and the proposed method is the feature ensemble part in the network. We argue that the adding more context features leads to a better skull stripping. We apply the exactly same pre-processing steps as described in Fig. 1 to all methods for the state-of-the-art comparison. For the UNet3D method, we re-implement the architecture and re-train CNN network using our dataset, which has exactly same data distribution as to the proposed method. However, for the DeepMedic method, we directly apply the pre-trained model to our data. An example case showing contours overlaid with the multiparametric sequence is shown in Fig. 3. The visualization skull stripping comparison is shown in Fig. 4 and the quantitative performance comparison is listed in Table 5. In addition, we also perform the analysis of variance (ANOVA) on dice score coefficient by comparing performances of these existing methods to our result, and the p-values are shown in the Table 5. All p-values are less than 0.001, which implies the proposed method providing a significant improvement on the skull stripping. The boxplot comparison of state-of-the-art is shown in Fig. 5.

**Table 5:** Skull stripping performance comparison to state-of-the-arts.

	# of cases	Dice	Precision	Recall	FPR	FNR	HD95 (mm)	p-value (t-test on Dice)
BET^23^	216	0.8494 ± 0.0455	0.7463 ± 0.0718	**0.9916** ± 0.0186	0.0650 ± 0.0224	**0.0084** ± 0.0186	19.9951 ± 5.4941	$9.19\times {10}^{-151}$
3dSS^47^	216	0.8427 ± 0.0449	0.7430 ± 0.0751	0.9809 ± 0.0279	0.0660 ± 0.0238	0.0191 ± 0.0279	19.9087 ± 4.4316	$1.29\times {10}^{-159}$
ROBEX^48^	216	0.9555 ± 0.0173	0.9730 ± 0.0236	0.9396 ± 0.0318	0.0053 ± 0.0057	0.0604 ± 0.0318	4.4792 ± 1.8869	$7.52\times {10}^{-54}$
UNet3D^38^	216	0.9773 ± 0.0179	0.9818 ± 0.0168	0.9735 ± 0.0290	0.0035 ± 0.0034	0.0265 ± 0.0290	3.1219 ± 2.7262	$5.61\times {10}^{-6}$
UPNN^39^	216	0.9743 ± 0.0257	0.9814 ± 0.0156	0.9684 ± 0.0405	0.0035 ± 0.0032	0.0316 ± 0.0405	3.3924 ± 2.8434	$4.22\times {10}^{-7}$
EnNet (ours)	216	**0.9850** ± 0.0171	**0.9940** ± 0.0093	0.9768 ± 0.0307	**0.0012** ± 0.0019	0.0232 ± 0.0307	**2.6098** ± 2.4814	–

The best result is highlighted in bold.

The performance comparison demonstrates that the proposed method offers the best results in terms of the dice, precision, recall, FPR, FNR, and the HD95. The small value of the standard deviation indicates the robustness of the skull stripping performance. We also notice an interesting thing: the BET has better performances on Recall and FPN, comparing to the proposed method. It may be that BET using T1 and T2 image modalities generates less false negatives. However, it produces lots of false positives.

## Discussion

Even though there are extensive works on skull stripping in literature^16,24,38–40^, to best of our knowledge, none of the methods/algorithms have explicitly quantitative analysis of performance on different image sequence combinations. It is known that different image provides different brain information, therefore, multiparametric MRIs are widely used in radiomics brain research, including brain segmentation, and brain tumor segmentation. In this work, we are the first group quantitatively showing the performance difference with different image sequence combinations.

**Figure 6:**
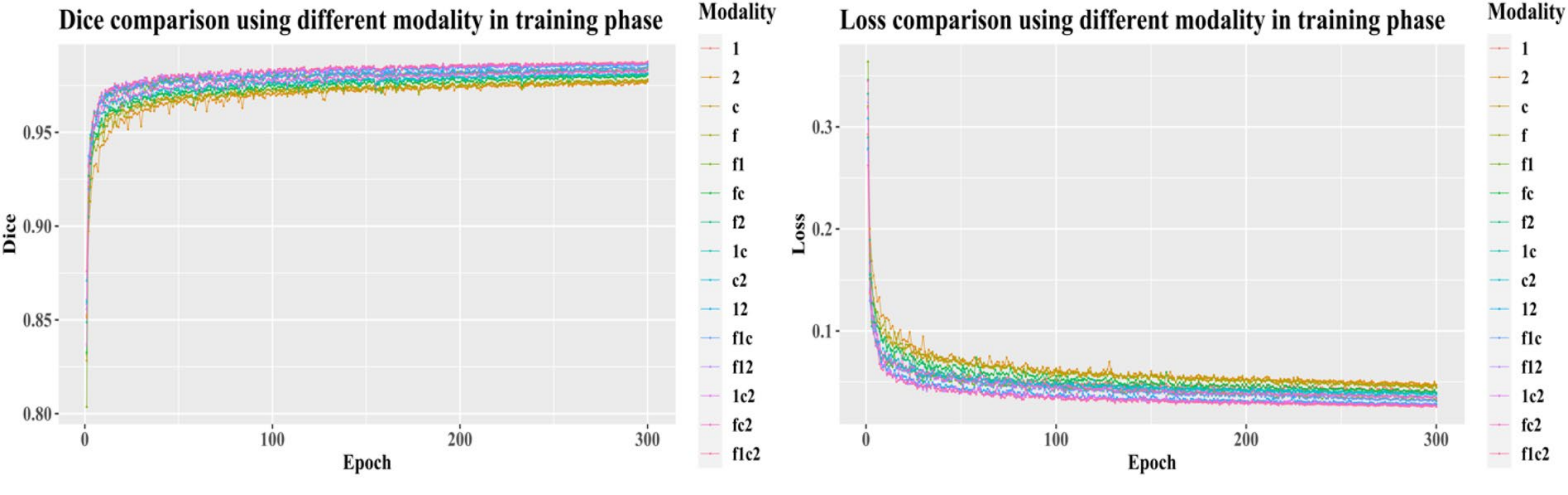
The change of dice (left) and loss (right) in the training phase. In the legend, 1, 2, c, and f represent T1, T2, T1ce, and T2-FLAIR, respectively. For example, f1ce represents that the combination has all four image sequences.

**Figure 7:**
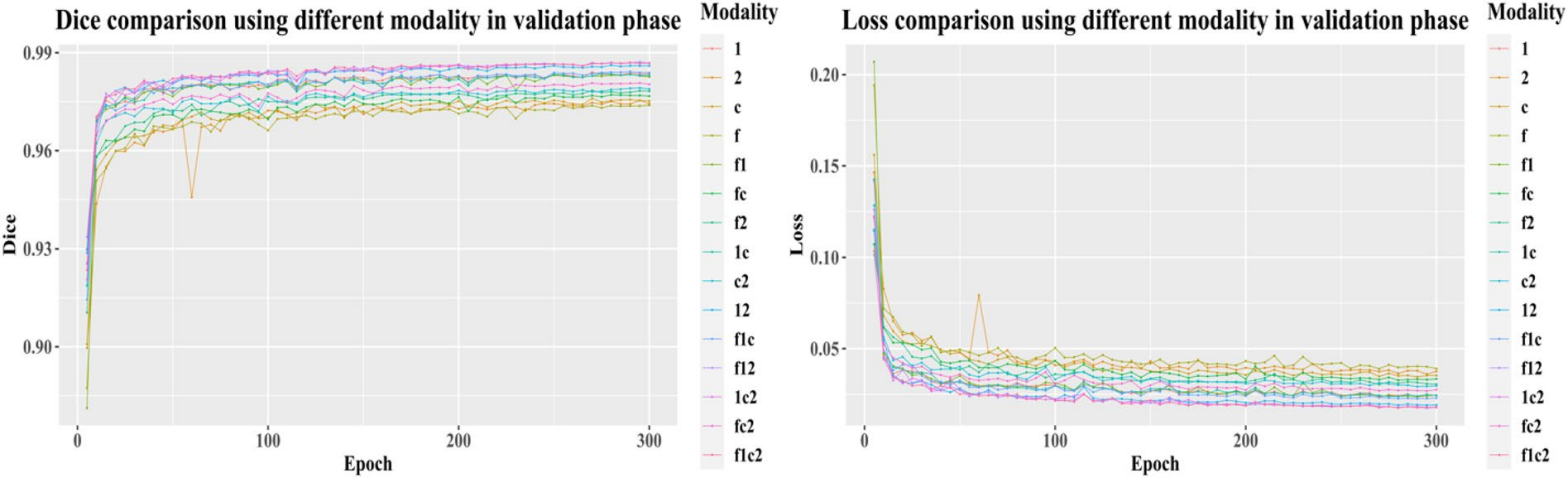
The change of dice (left) and loss (right) in the validation phase. To save training time, we execute the validation part in every 5 epochs. In the legend, 1, 2, c, and f represent T1, T2, T1ce, and T2-FLAIR, respectively. For example, f1ce means that the combination has all four image sequences.

To train the model, we randomly take 480 cases as the training dataset, and 119 cases as the validation dataset. We take the hyper-parameter setting as discussed in Section IV. The dice and loss change in the training phase and in the validation phase are plotted in Figs. 6 and 7. According to the result*,* it is easy to conclude that a combination of all four image sequences offers the best dice (0.9869 at epoch 300 in the validation phase) and least loss (0.0178 at epoch 300 in validation phase).

In the experiment, the average dice of skull stripping on the testing dataset is $0.985\pm 0.0171$. Considering the high mean dice of the performance with low standard deviation, it implies the proposed method offers a competitive and stable performance on brain extraction. In addition, we also investigate the model generality on different conditional MRIs, including healthy, pre-operative, and post-operative MRIs. The experimental result shows that the proposed method offers the best performance on pre-operative, which is most likely because the training data is coming from the pre-operative MRIs. However, there is no significant difference of the skull stripping between the healthy and post-operative MRIs as the p-value of the t-test is 0.3869. We further compare the performance of skull stripping on data from multi-centers. According to the experimental result, the performance of data from same center is significantly better (p-value of 0.0306) than that of different center because of the different scanner device parameters, or acquisition protocols.

We also notice an interesting result in the experiment. The state-of-the-art comparison shows that the BET has the best performance on recall and FNR. It may because the BET has lower false negatives compared to other methods.

**Figure 8:**
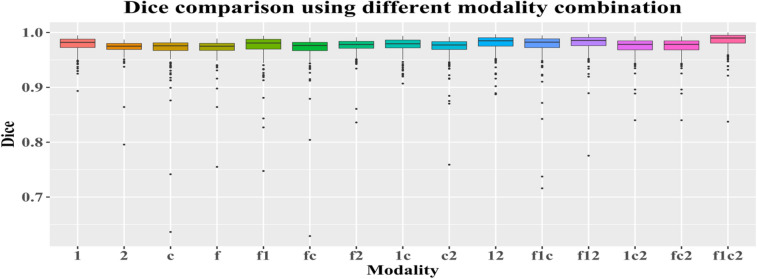
Quantitative dice comparison using different modality combination in the testing phase. In the x axis, 1, 2, c, and f representsT1, T2, T1ce, and T2-FLAIR, respectively. For an example, f1ce means that the combination has all four image sequences.

Furthermore, we also apply the models obtained from training with different modality combinations to quantitatively compare the skull stripping performance in the testing phase, and the result shows in Fig. 8. With integration of all multiparametric MRIs, the proposed convolutional neural network-based model which embeds ensemble features offers the best results.

Even though the proposed method provides a reliable and competitive performance on brain extraction, there are still some limitations. First, it requires a reliable co-registration for multi-parameters MRIs. Second, it has an underperformance on post-operative, specifically for cases with post-surgical cavity surgery close to outlier. The cavity may result in a poor performance. Third, source of image acquisition also impacts the skull stripping performance. To overcome the limitations, in future, we plan to increase more post-operative MRIs from multi-centers as the training data, and develop an advanced convolutional neural network model for the brain extraction.

## Conclusion

In this work, we propose a 3D convolutional neural network-based method to extract the brain. It is a fully automatic computer-aided method. The proposed method generally works for healthy brain MRIs, and pre-/post- operative brain MRIs with tumors as well. Moreover, the trained model using the proposed method is robust. It is not only applicable for in-house private data, but also for multicenter data. Comparing to the performance of state-of-the-art, the proposed method provides the best result. In addition, we first quantitatively evaluate the impact of skull stripping using different MRI sequences (combination). In future, we plan to increase more post-operative mpMRIs from multi-centers as the training data, and develop an advanced convolutional neural network model for the brain extraction.

## Data Availability

The partial datasets generated and/or analyzed during the current study are available in The Cancer Imaging Archive (TCIA) repository (link: https://www.cancerimagingarchive.net). The rest data are de-identified and privately owned by the University of Pittsburgh Medical Center (UPMC). To access the mpMRIs dataset from UPMC, please contact Dr. Colen.
