# Author Correction: A general skull stripping of multiparametric brain MRIs using 3D convolutional neural network

**DOI:** 10.1038/s41598-022-16725-y

**Published:** 2022-07-25

**Authors:** Linmin Pei, Murat Ak, Nourel Hoda M. Tahon, Serafettin Zenkin, Safa Alkarawi, Abdallah Kamal, Mahir Yilmaz, Lingling Chen, Mehmet Er, Nursima Ak, Rivka Colen

**Affiliations:** 1 Imaging and Visualization Group, ABCS, Frederick National Laboratory for Cancer Research, Frederick, MD 21702 USA; 2 Department of Radiology, University of Pittsburgh, Pittsburgh, PA 15260 USA; 3 Hillman Cancer Center, University of Pittsburgh Medical Center, Pittsburgh, PA 15232 USA

Correction to: *Scientific Reports* 10.1038/s41598-022-14983-4, published online 27 June 2022

The original version of this Article contained errors in the spelling of the authors Murat Ak and Nursima Ak, which were incorrectly given as A.K. Murat and A.K. Nursima, respectively.

The Author contribution statement now reads:

“L.P. designed and constructed the experiments and wrote the draft of the manuscript. M.A. and N.T. verified the ground truth of the experimental dataset and revised the manuscript. S.Z., S.A., A.K., M.Y., M.E. and N.A. verified the ground truth of the experimental dataset. L.C. revised the manuscript. R.C. supervised the whole project.”

The original Article has been corrected.

